# Persistent demographic differences in colorectal cancer screening utilization despite Medicare reimbursement

**DOI:** 10.1186/1471-230X-5-10

**Published:** 2005-03-08

**Authors:** Cynthia W Ko, William Kreuter, Laura-Mae Baldwin

**Affiliations:** 1 Department of Medicine, Division of Gastroenterology, Box 356424, University of Washington, Seattle, Washington, USA; 2 Department of Health Services, Center for Cost and Outcomes Research, Box 359736, University of Washington, Seattle, Washington, USA; 3 Department of Family Medicine, Box 354982, University of Washington, Seattle, Washington, USA

## Abstract

**Background:**

Colorectal cancer screening is widely recommended, but often under-utilized. In addition, significant demographic differences in screening utilization exist. Insurance coverage may be one factor influencing utilization of colorectal cancer screening tests.

**Methods:**

We conducted a retrospective analysis of claims for outpatient services for Washington state Medicare beneficiaries in calendar year 2000. We determined the proportion of beneficiaries utilizing screening fecal occult blood tests, flexible sigmoidoscopy, colonoscopy, or double contrast barium enema in the overall population and various demographic subgroups. Multiple logistic regression analysis was used to determine the relative odds of screening in different demographic groups.

**Results:**

Approximately 9.2% of beneficiaries had fecal occult blood tests, 7.2% had any colonoscopy, flexible sigmoidoscopy, or barium enema (invasive) colon tests, and 3.5% had invasive tests for screening indications. Colonoscopy accounted for 41% of all invasive tests for screening indications. Women were more likely to receive fecal occult blood test screening (OR 1.18; 95%CI 1.15, 1.21) and less likely to receive invasive tests for screening indications than men (OR 0.80, 95%CI 0.77, 0.83). Whites were more likely than other racial groups to receive any type of screening. Rural residents were more likely than urban residents to have fecal occult blood tests (OR 1.20, 95%CI 1.17, 1.23) but less likely to receive invasive tests for screening indications (OR 0.89; 95%CI 0.85, 0.93).

**Conclusion:**

Reported use of fecal occult blood testing remains modest. Overall use of the more invasive tests for screening indications remains essentially unchanged, but there has been a shift toward increased use of screening colonoscopy. Significant demographic differences in screening utilization persist despite consistent insurance coverage.

## Background

Screening for colorectal cancer is now recommended by several organizations [1-5], and insurance coverage of screening tests is becoming more widespread. For example, the Centers for Medicare and Medicaid Services began reimbursement for the commonly used screening tests in 1998, covering 100% of charges for fecal occult blood tests and 80% of charges for flexible sigmoidoscopy, colonoscopy for high risk individuals, and barium enema. Coverage was extended to include colonoscopy for average risk individuals in July, 2001. Despite existing guidelines, many eligible people are not receiving screening tests according to current recommendations [6-9]. In 2001, only 23.5% of surveyed adults over the age of 50 had received fecal occult blood testing in the previous year, and 43.4% had received lower endoscopy in the previous 10 years [7]. However, use of screening colonoscopy may be increasing [10]. Age, race, insurance coverage, and place of residence, have all been associated with utilization [7,9,11-15]. Although lack of insurance coverage may be one reason for under-utilization, we recently showed that the proportion of Medicare beneficiaries receiving invasive colorectal screening tests (defined as colonoscopy, flexible sigmoidoscopy, or barium enema) did not increase in 1998, immediately after introduction of Medicare coverage for these tests [16]. In a 9-month period during this year, only 6.3% of Washington state Medicare beneficiaries received fecal occult blood testing, 6.3% had any type of invasive tests, and 3.2% had invasive screening tests. The purpose of this study was to examine the effect of insurance coverage on overall utilization of screening tests and on demographic differences in screening utilization.

## Methods

### Data source

The study was approved by the University of Washington Institutional Review Board. We used the calendar year 2000 Physician/Supplier Part B Standard Analytic File and the Denominator File, which are administrative databases covering Medicare beneficiaries and maintained by the Centers for Medicare and Medicaid Services. The Denominator File contains information about date of birth, gender, race, place of residence, vital status, and enrollment in Medicare Part A, Medicare Part B or capitated health plans. The Physician/Supplier Part B Standard Analytic File contains claims data for outpatient physician and supplier services, including the date of the visit, associated diagnoses (coded as International Classification of Diseases [ICD9] codes), and procedures performed (coded as Current Procedural Terminology [CPT] or common procedure [HCPCS] codes).

### Patient selection

All Medicare beneficiaries listed as Washington State residents in the Denominator File in calendar year 2000 were eligible for inclusion (n = 772,153). Beneficiaries who were less than 65 years old (n = 132,711), who died during the study year (n = 32,678), or who were not enrolled in both parts A and B throughout the study year (n = 33,263) were excluded. We excluded patients enrolled in capitated health plans during any part of the study year (n = 170,232) because they may have received screening tests while in these plans for which claims were not submitted. Based on ICD9 codes in the Physician/Supplier Standard Analytical File, we also excluded patients with a diagnosis code for a personal history of colon polyps (V12.72, n = 683), colon or rectal cancer (V10.05 or V10.06, n = 398), or inflammatory bowel disease (555.x, 556.x, n = 227) in this calendar year, since these patients are at increased risk of colorectal cancer and may need more frequent surveillance. Patients without one of these diagnoses were analyzed as average risk. However, if patients did have a history of one of these conditions, but this code was not listed in calendar year 2000, they could have been misclassified as being at average risk. We did not exclude patients with a family history of colorectal cancer, as we felt they could not be reliably identified from the available ICD9 diagnosis codes. We had 401,961 eligible beneficiaries for analysis.

### Identification of screening tests

We identified screening fecal occult blood tests using the HCPCS code assigned by the Centers for Medicare and Medicaid Services for this test (G0107). We also examined the use of invasive colon tests (flexible sigmoidoscopy, colonoscopy, and double contrast barium enema). However, using ICD9 codes, it can be difficult to designate a given invasive colon test as screening or diagnostic [17]. To define tests as screening indication or diagnostic indication, we used the following algorithm, similar to our previous study [16]. We first identified these procedures using all CPT or HCPCS codes for colonoscopy, flexible sigmoidoscopy, and barium enema (colonoscopy – 44388, 44389, 44392, 44393, 44394, 45378, 45380, 45383, 45384, 45385, G0105, G0121; sigmoidoscopy – 45300, 45305, 45308, 45309, 45315, 45320, 45330, 45331, 45333, 45338, 45339, G0104; barium enema – 74270, 74280, G0106, G0120, G0122). We then defined an invasive procedure as performed for a screening indication if: 1) the procedure was coded using the relevant HCPCS codes for screening tests; 2) ICD9 codes V76.51 (screening-malignant neoplasm-colon) or V76.41 (screening-malignant neoplasm-rectum) were associated with the procedure; or 3) there were no ICD9 diagnosis codes of gastrointestinal tract symptoms, weight loss, or anemia associated with any physician visits within the previous 3 months (abdominal pain – 787.3, 789.0x, 789.6x; altered bowel habits – 564.0, 787.x; gastrointestinal bleeding – 578.x; positive fecal occult blood test – 792.1; weight loss – 783.2; iron deficiency anemia – 280.x; anemia, unspecified – 285.9). Because of this 3-month exclusion rule, we analyzed only claims submitted between April 1, 2000 and December 31, 2000. We analyzed only the first test performed, as later tests may have been performed to evaluate abnormalities found on the initial test.

### Data analysis

We determined the proportion of average risk beneficiaries who received screening fecal occult blood tests or who underwent flexible sigmoidoscopy, colonoscopy, or double contrast barium enema. For these tests, proportions were calculated using all tests identified by all CPT and/or HCPCS codes (all invasive tests) or only tests identified by the algorithm described above (invasive tests for screening indications).

We also analyzed screening test utilization in population subgroups as defined by age, sex, race, and place of residence (urban vs. rural). We compared differences in proportions of beneficiaries undergoing screening using chi-square tests. Place of residence was defined as urban or rural depending on the health service area in which the patient lived. Rural health service areas include all ZIP codes that are closest to a rural hospital, as defined by the Washington State Department of Health. Multiple logistic regression analysis was used to determine the relative odds of screening in different demographic groups (Stata 8.0, Stata Corp., College Station, TX). Significance of the regression models was tested using the log-likelihood statistic, and the method of Hosmer and Lemeshow was used to assess goodness of fit of the regression models.

## Results

Beneficiaries were predominantly white, and there were more females than males (Table 1). In the nine-month study period, 9.2% of Washington State Medicare beneficiaries had a claim submitted for screening fecal occult blood tests (Table 1). Fecal occult blood testing was more common in women than in men, in beneficiaries aged 70 to 74 than in other age groups, and in rural residents than in urban residents. Whites were the most likely to receive screening fecal occult blood tests, and Hispanics the least likely. These differences were all statistically significant (p < 0.001).

Overall, 7.2% had any invasive test (colonoscopy, flexible sigmoidoscopy, or barium enema for diagnostic or screening indications) in the 9-month study period (Table 1). Utilization of invasive tests for screening indications was uncommon, occurring in only 3.5% during the nine-month study period. With all invasive tests combined, men, beneficiaries aged 70 to 74, whites, and urban residents were more likely to utilize tests than women, other age groups, other racial groups, and rural residents, respectively. With all invasive tests for screening indications combined, similar demographic variation in utilization was found. Fifty-eight percent of all invasive tests and 41% of invasive tests for screening indications were colonoscopies.

However, when examining utilization of colonoscopy, sigmoidoscopy, and barium enema separately, some interesting demographic differences were seen (Table 1). Men, beneficiaries aged 70 to 74, whites, and urban residents were more likely to undergo colonoscopy. Flexible sigmoidoscopy was more common in men, beneficiaries age 65 to 69, whites, and urban residents. These differences were still present, but less pronounced when looking at colonoscopy and flexible sigmoidoscopy for screening indications. Use of barium enema for screening was infrequent in both rural and urban patients. Although Hispanics were less likely to utilize colonoscopy and sigmoidoscopy, they were more likely to undergo barium enema than whites.

We developed multiple logistic regression models to determine the relative odds of receiving screening tests in different population subgroups (Table 2). Parallel, previously published data from 1994–98 are presented for comparison [16]. These models show that women were more likely to receive screening fecal occult blood tests (odds ratio 1.18; 95% confidence interval 1.15, 1.21), but less like to receive invasive tests for screening indications (odds ratio 0.80; 95% confidence interval 0.77, 0.83). Beneficiaries aged 75 and over were less likely to be screened than younger beneficiaries. For example, compared with beneficiaries aged 65–69, those aged 75–79 were less likely to be screened with either fecal occult blood tests (odds ratio 0.94; 95% confidence interval 0.91, 0.96) or with invasive tests (odds ratio 0.82; 95% confidence interval 0.78, 0.86). Screening utilization was also significantly lower in beneficiaries aged 80 years or older compared with those aged 65–69. Hispanics were less likely than whites to be screened with either fecal occult blood tests (odds ratio 0.30; 95% confidence interval 0.23, 0.38) or with the invasive tests (odds ratio 0.40; 95% confidence interval 0.28, 0.56). Rural residents were more likely to be screened with fecal occult blood tests (odds ratio 1.20, 95% confidence interval 1.17, 1.23), but less likely to receive invasive tests for screening indications (odds ratio 0.89; 95% confidence interval 0.85, 0.93).

We developed similar multiple logistic regression models to look individually at utilization of colonoscopy, sigmoidoscopy, or barium enema for diagnostic or screening indications (Table 3). Utilization of colonoscopy and flexible sigmoidoscopy was less common in women than in men, while women were more likely to undergo barium enema (odds ratio 1.13, 95% confidence interval 1.04, 1.24). The odds of beneficiaries undergoing colonoscopy initially increased slightly with age, but then decreased at age 80 and over. The odds of undergoing sigmoidoscopy decreased with age, while the odds of undergoing barium enema increased with age. Hispanics were less likely than whites to undergo colonoscopy or sigmoidoscopy, but more likely to undergo barium enema (odds ratio 1.84, 95% confidence interval 1.22, 2.78). Other racial groups were less likely than whites to utilize colonoscopy, flexible sigmoidoscopy or barium enema. With inclusion of only screening tests (Table 4), women again utilized colonoscopy and sigmoidoscopy less often than men, but utilized barium enema similarly (odds ratio for barium enema 0.98, 95% confidence interval 0.84, 1.13). Utilization of colonoscopy was relatively constant until age 80, but then declined. Again, Hispanics were less likely than whites to utilize colonoscopy or sigmoidoscopy for screening indications (odds ratio for colonoscopy 0.36; 95% confidence interval 0.21, 0.62). Urban residents were more likely than rural residents to receive colonoscopy or sigmoidoscopy for screening indications (odds ratio for colonoscopy 1.10; 95% confidence interval 1.03, 1.18), but utilized barium enema similarly.

## Discussion

We previously showed that colorectal cancer screening tests are under-utilized and that utilization did not increase shortly after introduction of the Medicare screening benefit [16]. In this study, we extend these findings and examine the effect on utilization after 2 to 3 years of insurance coverage. Although utilization of fecal occult blood testing increased moderately between 1998 and 2000 (6.30% vs. 9.15% over 9 months, respectively), utilization of more invasive tests remained infrequent (6.26% in 1998 vs. 7.19% in 2000 receiving any invasive test; 3.17% in 1998 vs. 3.48% in 2000 receiving invasive tests for screening indications over 9 months). This was true for all demographic subgroups examined. However, there was some shift in the type of invasive procedure done, with increasing use of colonoscopy compared with flexible sigmoidoscopy and barium enema. In 2000, 58% of all invasive tests and 41% of invasive tests for screening indications were colonoscopies, compared to 47% and 35% in 1998, respectively. Medicare coverage for screening colonoscopy in average risk beneficiaries did not begin until July 2001, and therefore most colonoscopy exams during our study were likely done in high risk patients. Utilization of screening colonoscopy may have increased even further after this change in reimbursement policies to cover average risk individuals.

In addition, we show that insurance coverage for screening does not eliminate disparities in screening utilization. In fact, disparities actually increased over time in some instances. Compared to 1994–1998 [16], the relative odds of any invasive testing for Hispanics versus whites actually decreased in 2000, while the effect for invasive tests for screening indications in different racial groups was mixed (Table 2). Disparities related to gender and place of residence were essentially unchanged between 1994–8 and 2000. These findings extend those of other studies in the general population [12,14], where universal insurance coverage of screening was not present, and studies of previous years in Medicare beneficiaries [13,15].

The precise reasons for the observed demographic disparities in screening are unknown. The sex and race-related disparities are consistent with other data showing differential use of medical services in general in these population subgroups. Screening in general was most common in beneficiaries age 65 to 74. As the potential benefit of screening decreases with age and shorter life expectancy, the age-related decrement in screening after age 75 may be clinically appropriate. Regarding geographic differences, the availability of screening services, especially for the invasive and more resource intensive tests such as colonoscopy and sigmoidoscopy, may be greater in urban than in rural areas. Fecal occult blood testing is less resource intensive and is likely to be more available in rural areas, potentially explaining some of the geographic differences in screening.

Projecting these data out over a longer period gives a more complete picture of utilization differences. For example, 3.5% of whites, 2.8% of blacks, and 1.6% of Hispanics had invasive screening tests done over the 9-month study period. Assuming constant screening rates, over a 5-year period, 23% of whites would undergo invasive tests, compared to only 19% of blacks and 11% of Hispanics. These differences are further magnified if fecal occult blood test utilization is also considered. These screening disparities may contribute to the known differences in colorectal cancer incidence and survival in different racial groups [18,19].

This study has several limitations. First, we cannot clearly separate the effects of Medicare coverage from secular trends towards increasing utilization of screening tests. Second, we used administrative claims databases to assess health services utilization. Although the accuracy of coding for the diagnoses and procedures studied here is not established, claims coding surgical services and procedures is fairly reliable and accurate [20-24]. Third, we analyzed data from only one state, and these results may not necessarily be generalizable to other regions. In particular, the number of minorities in this study was relatively small, and the confidence intervals for minority groups in the multiple logistic regression models were wide. However, patterns of utilization of colorectal cancer screening tests were similar in Kansas Medicare beneficiaries [9,12]. Another study looking at national trends in colorectal tests in Medicare beneficiaries found that use of sigmoidoscopy, barium enema, and fecal occult blood testing declined over a similar period, while colonoscopy utilization increased [25].

Our study only included patients age 65 and older, and we did not assess utilization in patients younger than this. This was a cross-sectional study, and patients who may have previously been screened with procedures that are not recommended annually would have been classified as unscreened in our analysis. Utilization of colonoscopy and colonoscopy practice patterns may have changed substantially since 2000 [26-28]. Lastly, we excluded patients who enrolled in capitated health plans, where health services utilization patterns may differ from those in traditional fee-for-service plans [29,30]. Screening frequency should also be studied within these health plans.

Although we developed an algorithm to distinguish screening from diagnostic tests, we cannot be certain that tests we designated as screening were truly intended as screening tests. We excluded patients with physician visits for gastrointestinal symptoms over the prior 3 months, but it may take longer to have a colonoscopy scheduled for these indications. This may influence our estimates of screening frequency. In our previous study, we found that 82% of procedures designated as screening from the HCPCS codes would have been classified as screening from our algorithm [16]. However, even if all colonoscopies, flexible sigmoidoscopies, or barium enemas done were intended as screening, only 7.2% of the study population would have had an invasive screening test during the 9-month study period, or 9.6% per year. Since not all invasive tests done are intended for screening, we believe that less than 7% of our study population had invasive screening tests during the 9-month study period.

This study extends our previous work and shows that provision of insurance coverage of screening tests does not necessarily increase utilization of such tests in the medium term. Even 2 to 3 years after beginning universal coverage and widespread publicity about colorectal cancer screening [31], screening rates changed only modestly, and demographic differences in screening utilization remained. Thus, insurance coverage may be only one small factor affecting patients' decisions to undergo colorectal cancer screening [32]. We did find a moderate shift towards use of the most expensive test, colonoscopy, over time. It may be that the more ready availability of colonoscopy services in urban areas influences patients' or providers' decisions to use this form of screening. Conversely, female or non-white beneficiaries may be more reluctant to undergo invasive screening tests, or providers may be less likely to offer invasive tests to these subgroups. In addition, out-of-pocket costs for screening tests may still be prohibitive for some populations, affecting screening utilization. We did not have information about private insurance or indirect costs which could influence decisions about screening. These aspects of disparities in screening utilization cannot be addressed using administrative claims data. Therefore, further efforts should be made to identify and address additional barriers to and preferences about colorectal cancer screening in the general Medicare population, and especially in underserved subgroups.

## Conclusion

Overall utilization of colorectal cancer screening tests increased only modestly 2 to 3 years after institution of Medicare coverage, but there was a shift towards screening colonoscopy and away from less invasive tests. Demographic differences in screening persisted despite consistent insurance coverage.

## List of abbreviations

CPT: Current Procedural Terminology

HCPCS: Health Care Common Procedures Coding System

ICD9: International Classification of Diseases – 9

## Competing interests

The author(s) declare that they have no competing interests.

## Authors' contributions

CK conceived the study, participated in data analysis and drafted the manuscript. WK participated in design and analysis of the study. LMB participated in design of the study and data analysis. All authors read and approved the final manuscript.

## Pre-publication history

The pre-publication history for this paper can be accessed here:



## Figures and Tables

**Table 1:** Demographic characteristics of beneficiaries and proportion receiving colon tests

			Any invasive test *				Invasive screening test †			
	Demographic characteristics (n, %)	Fecal occult blood test (%)	All (%)	Colono- scopy (%)	Flexible sigmoid-oscopy (%)	Barium enema (%)	All (%)	Colon-oscopy (%)	Flexible sigmoid-oscopy (%)	Barium enema (%)

All subjects	401961 (100)	9.15	7.19	4.19	2.41	0.59	3.48	1.44	1.86	0.18

Sex										
Male	168206 (41.8)	8.57*	7.48*	4.31*	2.64*	0.54*	4.04*	1.73*	2.12*	0.18
Female	233755 (58.2)	9.57	6.97	4.11	2.25	0.62	3.08	1.23	1.66	0.18

Age										
65–69	100754 (25.1)	10.27*	7.87*	4.35*	3.05*	0.47*	4.39*	1.71*	2.51*	0.18
70–74	100454 (25.0)	10.39	8.04	4.59	2.87	0.59	4.19	1.73	2.27	0.19
75–79	90975 (22.6)	9.77	7.56	4.53	2.39	0.64	3.60	1.58	1.82	0.20
≥ 80	109778 (27.3)	6.48	5.46	3.39	1.41	0.65	1.89	0.83	0.90	0.16

Race										
White	380492 (94.7)	9.36*	7.27*	4.23*	2.45*	0.59*	3.53*	1.46*	1.89*	0.18
Black	5649 (1.4)	4.66	6.30	3.77	2.05	0.48	2.81	1.26	1.49	0.07
Hispanic	2179 (0.5)	3.12	4.68	2.75	0.87	1.06	1.61	0.60	0.69	0.32
Asian	6946 (1.7)	5.83	5.64	3.43	1.86	0.30	2.58	1.04	1.41	0.13
Other	6595 (1.6)	6.60	5.78	3.71	1.77	0.29	2.56	1.12	1.35	0.09

Residence										
Rural	73041 (18.2)	10.64*	6.85*	4.04	2.28	0.54	3.26*	1.37	1.72*	0.17
Urban	328920 (81.8)	8.82	7.26	4.22	2.44	0.60	3.53	1.46	1.89	0.18

* Statistically significant difference (p < 0.005).

* All flexible sigmoidoscopy, colonoscopy, or barium enema exams

† Invasive tests (flexible sigmoidoscopy, colonoscopy, or barium enema) without an associated exclusion diagnosis such as abdominal pain or anemia

**Table 2:** Multivariable models of characteristics associated with utilization of colon tests

	OR (95% CI) for fecal occult blood test, year 2000	OR (95% CI) for any invasive test*, 1994–1998	OR (95% CI) for any invasive test, year 2000	OR (95% CI) for screening colon test*, 1994–1998	OR (95% CI) for screening colon test year 2000
Sex					
Male	1.00	1.00	1.00	1.00	1.00
Female	1.18 (1.15, 1.21)	0.99 (0.98, 1.01)	0.96 (0.93, 0.98)	0.84 (0.82, 0.86)	0.80 (0.77, 0.83)

Age					
65–69 yrs	1.00	1.00	1.00	1.00	1.00
70–74 yrs	1.01 (0.98. 1.04)	1.12 (1.10, 1.14)	1.09 (0.99, 1.06)	1.05 (1.02, 1.08)	0.96 (0.92, 1.00)
75–79 yrs	0.94 (0.91, 0.96)	1.14 (1.12, 1.17)	0.96 (0.92, 0.99)	0.99 (0.96, 1.01)	0.82 (0.78, 0.86)
≥ 80 yrs	0.59 (0.57, 0.61)	0.83 (0.82, 0.85)	0.68 (0.65, 0.70)	0.60 (0.58, 0.61)	0.43 (0.41, 0.45)

Race					
White	1.00	1.00	1.00	1.00	1.00
Black	0.47 (0.42, 0.54)	0.83 (0.78, 0.89)	0.83 (0.74, 0.92)	0.68 (0.62, 0.76)	0.74 (0.63, 0.87)
Hispanic	0.30 (0.23, 0.38)	0.65 (0.56, 0.76)	0.59 (0.48, 0.72)	0.48 (0.38, 0.61)	0.40 (0.28, 0.56)
Asian	0.60 (0.54, 0.66)	0.72 (0.66, 0.78)	0.74 (0.67, 0.82)	0.65 (0.58, 0.72)	0.70 (0.60, 0.81)
Other	0.67 (0.61, 0.74)	0.71 (0.68, 0.75)	0.77 (0.69, 0.85)	0.66 (0.62, 0.71)	0.40 (0.28, 0.56)

Residence					
Urban	1.00	1.00	1.00	1.00	1.00
Rural	1.20 (1.17, 1.23)	0.94 (0.92, 0.96)	0.92 (0.89, 0.95)	0.92 (0.89, 0.94)	0.89 (0.85, 0.93)

* Data from 1994–98 was published previously [16].

**Table 3:** Multivariable models of characteristics associated with utilization of any colonoscopy, flexible sigmoidoscopy, or barium enema

	Colonoscopy		Flexible sigmoidoscopy		Barium enema	
	
	Odds ratio	95% confidence interval	Odds ratio	95% confidence interval	Odds ratio	95% confidence interval
	
Sex						
Male	1.00		1.00		1.00	
Female	0.97	0.97, 1.00	0.89	0.86, 0.93	1.13	1.04, 1.24
Age						
65–69	1.00		1.00		1.00	
70–74	1.06	1.01, 1.10	0.94	0.89, 0.99	1.25	1.10, 1.41
75–79	1.04	1.00, 1.08	0.78	0.74, 0.82	1.35	1.19, 1.52
≥ 80	0.77	0.74, 0.81	0.46	0.43, 0.49	1.38	1.22, 1.55

Race						
White	1.00		1.00		1.00	
Black	0.87	0.76, 0.99	0.79	0.66, 0.95	0.81	0.55, 1.18
Hispanic	0.61	0.48, 0.80	0.32	0.20, 0.50	1.84	1.22, 2.78
Asian	0.78	0.69, 0.89	0.73	0.62, 0.87	0.59	0.40, 0.87
Other	0.87	0.76, 0.99	0.69	0.58, 0.83	0.49	0.31, 0.77

Residence						
Rural	1.00		1.00		1.00	
Urban	1.06	1.02, 1.11	1.10	1.05, 1.16	1.11	0.99, 1.24

**Table 4:** Multivariable models of characteristics associated with utilization of screening colonoscopy, flexible sigmoidoscopy, or barium enema

	Colonoscopy		Flexible sigmoidoscopy		Barium enema	
	
	Odds ratio	95% confidence interval	Odds ratio	95% confidence interval	Odds ratio	95% confidence interval
	
Sex						
Male	1.00		1.00		1.00	
Female	0.74	0.71, 0.78	0.84	0.80, 0.88	0.98	0.84, 1.13
Age						
65–69	1.00		1.00		1.00	
70–74	1.02	0.96, 1.09	0.91	0.86, 0.96	1.06	0.86, 1.30
75–79	0.94	0.87, 1.01	0.73	0.68, 0.77	1.13	0.92, 1.40
≥ 80	0.49	0.46, 0.54	0.36	0.33, 0.39	0.90	0.73, 1.11

Race						
White	1.00		1.00		1.00	
Black	0.81	0.64, 1.03	0.73	0.59, 0.91	0.38	0.14, 1.02
Hispanic	0.36	0.21, 0.62	0.32	0.19, 0.53	1.72	0.82, 3.63
Asian	0.69	0.54, 0.86	0.72	0.59, 0.88	0.69	0.36, 1.34
Other	0.75	0.60, 0.95	0.68	0.55, 0.89	0.50	0.22, 1.11

Residence						
Rural	1.10		1.00		1.00	
Urban	1.10	1.03, 1.18	1.14	1.05, 1.18	1.09	0.90, 1.32
